# Perceptions of Polymethyl Methacrylate Cement Exposure Among Female Orthopaedic Surgeons

**DOI:** 10.5435/JAAOSGlobal-D-19-00117

**Published:** 2020-03-02

**Authors:** Katharine D. Harper, Rachel Bratescu, David Dong, Stephen J. Incavo, Shari R. Liberman

**Affiliations:** From the Department of Orthopedics and Sports Medicine, Houston Methodist Hospital, Houston, TX.

## Abstract

The medical field has long believed that polymethyl methacrylate (PMMA) vapor is dangerous to a growing fetus, and therefore, women who are pregnant should avoid exposure to it. Symptoms of vapor exposure include eye irritation, coughing, respiratory tract irritation, and irritation of exposed mucous membranes. The purpose of this study is to investigate the perceptions of PMMA cement exposure during pregnancy in female orthopaedic surgeons because it influences (1) the currently held beliefs and practices and (2) clinical and career choices.

**Methods:**

A 23-question survey was distributed via e-mail to all active members of the Ruth Jackson Orthopaedic Society and the “Women in Orthopaedics” private Facebook group. Questions consisted of the level of training, current usage of PMMA, previous exposure during pregnancy and/or breastfeeding, and beliefs regarding current or future willingness of exposure during pregnancy/breastfeeding.

**Results:**

PMMA training was found to have a positive correlation with those who remained in the operating room while pregnant or would do so in the future. Overall responses found that 41.7% would leave the room in the future if PMMA were being used while they were pregnant, with 24.7% leaving if they were breastfeeding. If they were the primary surgeon, 23.7% stated that they would leave and 8.4% stated that PMMA exposure during pregnancy factored into which subspecialty they chose.

**Conclusion:**

This survey demonstrates a lack of consensus among practicing female orthopaedic surgeons regarding the risks posed by remaining in a room during pregnancy and breastfeeding while PMMA is in use. Currently held beliefs and education practices should be examined to determine if they match the available literature.

The World Health Organization describes a polymethyl methacrylate (PMMA) compound as a colorless, volatile liquid with multiple applications, including as a bonding agent. It is a commonly used polymer in orthopaedic surgery, with particular prevalence in total joint arthroplasty and trauma.^1^ The belief among the medical field has long been held that exposure to vapors of this polymer is dangerous to a growing fetus, and as such, women who are pregnant should avoid exposure to it.^2,3^ Symptoms of vapor exposure include eye irritation, coughing, respiratory tract irritation, and irritation of exposed mucous membranes.^1^ The official Federal Drug Administration position on PMMA is that it is a class II device (moderate risk to a patient and/or user). This was downgraded from a class III device (high risk to a patient and/or user) in 1998.^4,5^ No studies exist on human embryos, and the Federal Drug Administration states that safety and effectiveness in pregnant women is not established.^4^ Although the American College of Obstetricians & Gynecologists do not have an apposition statement specifically targeting PMMA, they do have a policy regarding general workplace teratogen exposure. They state that “If exposures are identified, patients can be educated regarding the avoidance of exposure to toxic agents and, when necessary, referred to occupational medicine programs.”^6^ The purpose of this study is to investigate the perceptions of PMMA cement exposure during pregnancy in female orthopaedic surgeons because it influences (1) the currently held beliefs and practices and (2) whether these contribute to clinical and career choices.

## Methods

This cross-sectional study was performed from December 2018 to May of 2019, centered out of an urban, tertiary referral center. A 23-question survey was created and remained open for response for 5 months, with content based on demographics and perceptions regarding PMMA exposure during pregnancy and breastfeeding. The questions were determined within the research group after brainstorming sessions, and full beta testing by our departmental research department was done to ensure appropriate survey flow. The duration of survey was determined before release and was decided to remain open for 1 month after the final notification from the Ruth Jackson Orthopaedic Society (RJOS), based on previous response rate experience within the department. The survey was designed, reviewed by the authors, and distributed via e-mail to all active members of the RJOS, and also a private internet-based social media platform group for women in the field of orthopaedics (“Women in Orthopedics,” private Facebook© group). Permission was acquired from both groups' leadership to distribute the survey. The members were informed to only answer the survey once if they received the distribution from both sources. The questions consisted of age and the level of training, current usage of PMMA in practice, previous exposure during pregnancy and/or breastfeeding, and beliefs regarding the current or future willingness of exposure during pregnancy. Finally, questions regarding whether they had received training regarding PMMA and if their beliefs regarding PMMA influenced their specialty choice (see complete survey Appendix 1, Supplemental Digital Content, http://links.lww.com/JG9/A66). Some questions were only answered by some survey takers, based on previous responses to questions, where the survey flow allowed questions regarding pregnancy and/or lactation to be skipped if respondents had never experienced a pregnancy. Inclusion criteria were cognizant of the society's and groups' requirements through which the survey was distributed. Practicing orthopaedic surgeons (either in residency/fellowship training or in independent practice) and retired surgeons were included, with instructions to the RJOS to distribute to e-mails of physicians who fit these criteria. Exclusion criteria included incomplete surveys. The results were collected via SurveyMonkey©, with a disclosure that results of the survey would be reported on anonymously. Spearman rank correlation coefficient was used to assess statistical dependence between survey responses. No external source of funding was received for this study.

## Results

Two hundred seventy-eight survey responses were received, of which 256 met the inclusion criteria. The RJOS membership is reported to be approximately 650, whereas the private social media platform had approximately 1,000 members at the time of survey distribution. This gives us a response rate of 25.5% from the RJOS (166 of 278 responses) and 11% from the private social media group (112 of 278 responses), with an overall response rate of 16.8% (278 of 1,650). We excluded incomplete surveys (22). Approximately 48% of respondents were between 35 and 44 years of age, whereas 73.5% were at an attending-level of practice. Greater than 70% currently use PMMA in training/practice, and >90% of survey respondents reported awareness of risks surrounding PMMA in pregnancy.

**Figure 1:**
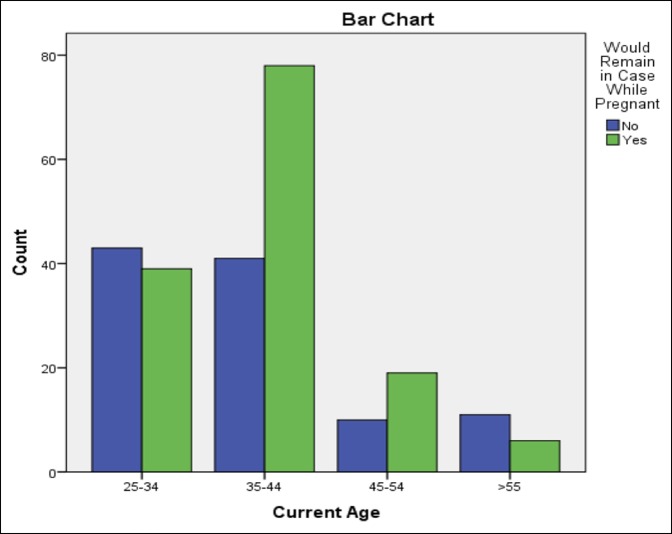
Chart demonstrating the survey responses regarding whether a surgeon would remain in a room using polymethyl methacrylate while pregnant, broken down by age of respondent.

**Figure 2:**
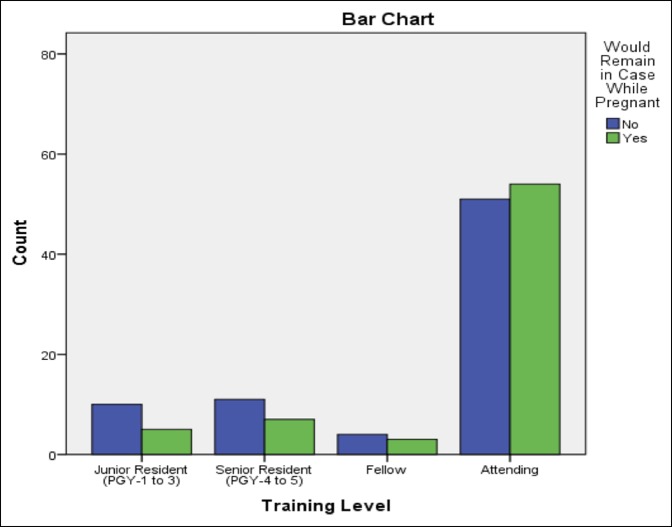
Chart demonstrating survey responses regarding whether a surgeon would remain in a room using polymethyl methacrylate while pregnant, broken down by training level.

The comparison analysis results are summarized in Table 1. Respondents who were older than 35 years of age were more likely to state that they would remain in a room using PMMA while pregnant in the future (*P* = 0.013) but did not correlate with those who actually remained in the room (*P* = 0.42) (Figure 1). PMMA training was found to have a positive correlation with those who remained in the operating room in the past while pregnant (odds ratio [OR] = 2.95, *P* = 0.023) or would do so in the future (OR = 2.72, *P* = 0.002). Respondents who currently used PMMA in practice were more likely to state they would remain in a case in the future (OR = 2.69, *P* = 0.001). If a respondent had previously been exposed to PMMA while breastfeeding, they would be more likely to state that they would remain in a room in the future while pregnant or breastfeeding (OR = 4.8, *P* = < 0.0001). The level of training was not shown to have an effect on whether respondents chose to leave or stay (Figure 2).

**Table 1:** Comparison Analysis Results From the Survey With Associated Statistical Significance

Analyzed Factor	Responses (n)	Comparison	OR	95% CI	*P* value
Received PMMA training	139	While pregnant, has remained in case	2.95	1.13-7.73	0.023
Received PMMA training	248	If pregnant, would remain in case	2.72	1.42-5.23	0.002
Has covered case using PMMA	246	If pregnant, would remain in case	4.47	2.61-7.68	<0.0001
Currently use PMMA in practice	248	If pregnant, would remain in case	2.69	1.52-4.77	0.001
Had exposure while breastfeeding	241	If pregnant, would remain in case	4.8	2.70-8.51	<0.0001

OR = odds ratio, PMMA = polymethyl methacrylate.

Interestingly, an increased number of respondents (41.7%) stated that they would leave the room in the future if PMMA was being used while they were pregnant than those whom had actually left the room, with an additional 24.7% leaving in the future if they were breastfeeding. In addition, 23.7% stated they would leave the room if they were the primary surgeon on the case and 8.4% of female surgeons admitted that PMMA exposure during pregnancy factored into which subspecialty they chose.

## Discussion

It is difficult to conclude what knowledge orthopaedic surgeons currently possess on exposure risks with PMMA, particularly during pregnancy. Although most female surgeons (>90%) state that they are aware of the risks, it is difficult to ascertain true knowledge. Therefore, in brief, PMMA is a colorless, volatile liquid with distinct odor, high vapor pressure (4 kPa at 20°C), and moderate water solubility (15.8 g/L). The purity of commercial methyl methacrylate is typically 99.9%.^1,7^ PMMA vapor released into the air is broken down by exposure to hydroxyl radicals and ozone.^8^ The reported half-life of this process is 15 hours (hydroxyl) and 1 day (ozone), respectively.^8-10^ Interestingly, the majority (90%) of surgeons who left the room stated that they did so during mixing only (17%) or from the start of mixing to cement hardening (73%). This time frame is considerably less than a single half-life of the vapor; therefore, it is difficult to determine what benefit, if any, was the regarding exposure.

The literature regarding fetal toxicity and teratogenicity after exposure to PMMA vapor is limited. A study by Mclaughlin et al^11^ published in 1978 is one of the most comprehensive available finding that continuous exposure of 2 hours twice/d in pregnant mice from day 6 to 15 of gestation resulted in no fetal abnormalities. Exposures in this study were measured to be 1,330 ppm. An additional study by Solomon et al in 1993 where pregnant rats were exposed to PMMA vapors for 6 h/d from day 6 to 15 gestation found no fetal abnormalities. However, this study did find decreased maternal body weight with all exposure levels (ranging from 99 ppm up to 2028 ppm).^12^ Neither of these early studies did PMMA mixing in a vacuum setting. Earlier studies where PMMA exposure resulted in maternal toxicity (exposure levels were unreported) did show evidence of fetal abnormalities or death,^13,14^ but per the World Health Organization, these studies were not adequately reported to consider in the data.^1^ The current available literature finds that if maternal toxicity is not achieved (resulting in maternal symptoms including mucous membrane irritation, nausea, and respiratory irritation^1^), then fetal exposure does not result in teratogenicity or fetal toxicity.^14,15^ Additional studies evaluating fertility after exposure to PMMA have found no effect on the ability to conceive in mice (with exposures from 100 to 9000 ppm).^16^

Studies evaluating lactating surgeon exposure and breast milk PMMA concentrations have found no evidence of increased levels of methyl methacrylate within the breast milk after exposure.^3,17^ A study on lactating surgeons doing total joint arthroplasty found <0.5 ppm in the serum and breast milk, with surgeon samples not testing at a higher level than the control specimens.^17^

Without the context of typical exposure values in a modern-day operating room, these results are hard to interpret. The RJOS recently put out a position statement on exposure to PMMA in the operating room after the distribution and completion of our survey. It stated that “In cases where an adequately trained staff member is available, cement mixing should be performed by a non-pregnant team member.” However, a study by Schlegal et al^18^ evaluated seven different PMMA mixing techniques and found that no vapor exposure value increased above 25 ppm, with the four vacuum-mixed techniques staying below 10 ppm. They concluded that even with repeat mixing throughout the day, it did not constitute an increased health risk, particularly if vacuum mixing was used. A study by Jelecevic et al^19^ evaluated different vacuum mixing systems for exposure amounts and found the highest concentration achieved was 7.98 ppm. These studies also determined that using a vacuum-mixing system decreased exposure by between 50% and 75% compared with hand-mixing techniques.^18,19^ In real world application, monitors were worn by surgeons doing vertebroplasty and found their cumulative exposure for 1 hour was 4.8 ppm.^20^ They concluded that extrapolating that to an 8-hour, or even a 12-hour, workday would keep surgeon exposures acceptable,^20^ well below the Occupational Safety & Health Administrations recommended 100 ppm per day.^19,20^ These exposure values are also well below the previously done fetal teratogenicity studies. This is a particularly important distinction in total joint arthroplasty cases, where cement mixing and placement of implants is considered a vital portion of the procedure. It is difficult to determine which adequately trained staff member may be deemed to be with such an important part of the case. With nearly one-fourth of respondents stating that they would leave the room if they were the primary surgeon on the case, there are also implications with the need to disclose an absence to patients if this was to occur during a total joint arthroplasty.

The professional and social implications of the beliefs demonstrated here are far-reaching. 8.4% of respondents admitted that concern regarding PMMA exposure during pregnancy influenced which subspecialty they selected. For specialties that commonly use PMMA in their most common procedures (adult reconstruction and trauma), this could be a barrier to them attracting more females to the specialty. Female membership in the American Association of Hip and Knee Surgeons, the largest adult reconstruction society in the United States, amounts to just 2.7% of total membership (62 women), with similarly low numbers in the Knee Society (0.5%) and Hip Society (0.6%).^21^ The Orthopedic Trauma Association has a female distribution of 13% active membership, although only 3.8% of all membership types are made up of women.

Educating all orthopaedic surgeons on the true reported risks of PMMA during pregnancy will help dissuade the spread of misinformation. Misinformed trainees and attending surgeons may feel forced into revealing pregnancies earlier than desired because of fear of exposure. Indeed, in our study, we found a correlation to PMMA education leading to the surgeon being more likely to stay in a room using it during pregnancy, pointing us toward a trend that accurate education can perhaps dissuade some fears. With >40% of respondents stating that they would not stay in a room while pregnant in the future, this education is vitally important. In addition, by improving education, we can hopefully encourage more women to seek out subspecialties with regular PMMA exposure at a more frequent rate. The issues experienced here certainly extend beyond female orthopaedic surgeons. As leaders in the operating room, female support staff may look to female (and male) surgeons to help guide their decisions regarding exposure, and therefore, efforts to educate all surgeons (male and female) and reach a consensus should be put forth.

Limitations for the study include the limited data points available for interpretation and selection bias in the way the survey was distributed and answered. The response rates are considered average for externally distributed surveys,^22-24^ but it should be noted that large population crossover between the two groups exist. In addition, we are unable to determine how many of the members on the social media platform interact regularly with the platform in question; therefore, the actual response rates would likely be higher than the numbers reported here. Most respondents (>95%) were from the United States and Canada, and therefore, cultural biases may exist in the response to this survey. Although 90% of respondents claimed that they were aware of the literature surrounding PMMA, we did not evaluate if their current knowledge base is accurate, concurring with what is available in the scientific literature. More than 70% of respondents reported current use of PMMA in practice. People more likely to use PMMA may have been more likely to answer the survey and therefore potentially be more knowledgeable on the compound, skewing responses. Response bias may exist for those who have previously exposed themselves and experienced no ill effects, and therefore, an optimal survey would be in those female surgeons who have not yet had to make such a decision. Finally, respondents' true knowledge of PMMA was not able to be determined from the survey nor were the reasons for decisions able to be derived from our simple-response survey, making it difficult to determine the causation of the beliefs reported here.

## Conclusion

This survey demonstrates a lack of consensus among practicing female orthopaedic surgeons regarding the risks of exposure to PMMA during pregnancy and breastfeeding. The currently available literature on PMMA exposure during pregnancy and breastfeeding suggest no risk to the fetus or breastfeeding baby. Despite 90% of respondents claiming that they are aware of the risks of PMMA, 40% of respondents stated they would leave the room while pregnant in the future, leading us to think that the currently held beliefs and education practices should be examined to determine if they match the available literature. It is plausible that beliefs regarding this exposure are deterring individuals from pursuing specialties where PMMA is used regularly, with some 8.4% of respondents confirming as such. Additional epidemiologic, clinical, and animal-based studies are necessary to evaluate the true risk in modern-age cement techniques to allow for a consensus on workplace exposure and safety.
